# The Myopic Normal Tension Glaucoma Treatment Study (MYNT-GTS): Protocol and Baseline Characteristics of a Randomized Controlled Trial

**DOI:** 10.3390/jcm15197734

**Published:** 2026-10-08

**Authors:** Eun Ji Lee, Joon Mo Kim, Jiwoong Lee, Sangwoo Moon, Sang Woo Park, Mi Sun Sung, Ji-Ah Kim, Gyu-Nam Kim, Jong Chul Han, Ji Eun Song, Kyoung Min Lee, Tae-Woo Kim

**Affiliations:** 1 Department of Ophthalmology, Seoul National University College of Medicine, Seoul National University Bundang Hospital, Seongnam 13620, Republic of Korea; opticdisc@gmail.com; 2 Department of Ophthalmology, Kangbuk Samsung Hospital, Sungkyunkwan University School of Medicine, Seoul 03181, Republic of Korea; kjoonmo1@gmail.com; 3 Department of Ophthalmology, Pusan National University College of Medicine, Pusan National University Hospital, Busan 49241, Republic of Korea; glaucoma@pusan.ac.kr; 4 Department of Ophthalmology, Pusan National University College of Medicine, Pusan National University Yangsan Hospital, Yangsan 50612, Republic of Korea; anstkddn0421@hanmail.net; 5 Department of Ophthalmology, Chonnam National University Hospital, Gwangju 61469, Republic of Korea; exo70@naver.com (S.W.P.); sms84831@hanmail.net (M.S.S.); 6 Department of Ophthalmology, Ewha Womans University College of Medicine, Ewha Womans University Seoul Hospital, Seoul 07804, Republic of Korea; jiah423@gmail.com; 7 Department of Ophthalmology, Institute of Health Sciences, Gyeongsang National University School of Medicine, Gyeongsang National University Hospital, Jinju 52727, Republic of Korea; sayuh@naver.com; 8 Department of Ophthalmology, Samsung Medical Center, Sungkyunkwan University School of Medicine, Seoul 06351, Republic of Korea; heartmedic79@gmail.com; 9 Department of Ophthalmology, Kangwon National University School of Medicine, Chuncheon 24341, Republic of Korea; zdms222255@gmail.com; 10 Department of Ophthalmology, Seoul National University Boramae Medical Center, Seoul 07061, Republic of Korea; isletzz@gmail.com

**Keywords:** myopia, normal-tension glaucoma, intraocular pressure, randomized controlled trial

## Abstract

**Background/Objectives**: To describe the study protocol and baseline characteristics of participants in the Myopic Normal-Tension Glaucoma Treatment Study (MYNT-GTS), which is a randomized controlled trial evaluating the effect of intraocular pressure (IOP)-lowering treatment on glaucoma progression in eyes with myopic normal-tension glaucoma (NTG). **Methods**: A prospective, multicenter, randomized controlled clinical trial and baseline cross-sectional analysis. The study enrolled 473 treatment-naïve NTG eyes with myopia (spherical equivalent ≤ −1.0 diopters or axial length [AXL] > 24.0 mm) aged 20–60 years from 9 tertiary referral centers in South Korea. Participants were randomized at a 1:1 ratio into a treated group and an untreated group managed by active surveillance, and stratified by age, baseline IOP, AXL, and visual field (VF) mean deviation (MD). Treatment was initiated or intensified when predefined structural or functional progression was detected. Comprehensive examinations including quantitative assessments of the optic nerve head structure and microvasculature, medication adherence, and quality of life were performed every 4 months during the first year and every 6 months thereafter. Glaucoma progression was assessed from the rate of circumpapillary retinal nerve fiber layer (RNFL) thinning, and the rate of VF deterioration was assessed from the MD or visual field index (VFI). **Results**: The 473 enrolled participants comprised 239 assigned to the treated group and 234 to the untreated group. Most of the baseline variables did not differ significantly between the groups, including age (43.1 ± 9.1 vs. 43.9 ± 9.1 years [mean ± standard deviation], *p* = 0.33), baseline IOP (14.9 ± 2.6 vs. 14.7 ± 2.5 mmHg, *p* = 0.26), AXL (26.31 ± 1.37 vs. 26.32 ± 1.34 mm, *p* = 0.94), global RNFL thickness (79.5 ± 11.4 vs. 80.5 ± 13.2 μm, *p* = 0.38), and VF MD (−2.71 ± 2.58 vs. −2.32 ± 2.52 dB, *p* = 0.10). **Conclusions**: The vast majority of the analyzed variables did not differ significantly between the treated and untreated groups at baseline, supporting the validity of the subsequent comparisons. As the first prospective, multicenter randomized controlled trial dedicated to myopic NTG, the MYNT-GTS should clarify the efficacy of IOP-lowering treatment and inform the individualized management of myopic NTG. **Trial Registration**: Clinical Research Information Service (CRIS), Republic of Korea, KCT0005225.

## 1. Introduction

Glaucoma is a multifactorial disease, and this complexity is especially evident in normal-tension glaucoma (NTG), which can develop and progress despite relatively low intraocular pressure (IOP). NTG accounts for more than 70% of all glaucoma cases in Korea [1], which is a notably high proportion. While elevated pressure and ischemia have traditionally been regarded as principal mechanisms of NTG pathogenesis, myopia has recently emerged as an additional, clinically important factor that co-occurs in a substantial proportion of NTG patients [2,3,4,5]. Although myopia is a well-established risk factor for glaucoma onset, there is recent evidence that progression may actually be slower in myopic than nonmyopic eyes [6,7,8,9], while progression can be faster in younger patients with myopic NTG [10].

In myopia, axial elongation imposes tensile stress on the peripapillary sclera and the lamina cribrosa (LC) that induces structural deformation of the optic disc and parapapillary tissues that is thought to contribute to glaucoma susceptibility [6,11,12]. Because this damage appears to arise largely from myopia-related mechanical remodeling rather than solely from an elevated IOP, it has been regarded by some as a distinct entity, termed “myopic optic neuropathy” [13,14]. Several clinical features support this notion: glaucomatous damage in myopic eyes tends to occur at a younger age and at a normal IOP [6,9], and it is frequently aligned with the direction of optic disc torsion [15] and—particularly when concordant with the disc deformation—often shows little progression during long-term follow-up [16,17], with many eyes remaining stable even in the absence of IOP-lowering treatment [7,9]. Moreover, axial elongation generally stops after adolescence, so that once globe growth is complete the mature eye may be subjected to less ongoing mechanical stress [6,7,8]. Together these observations suggest that, despite similar patterns of optic nerve head (ONH) damage, the pathogenesis of “myopic glaucoma” differs from that of typical glaucoma, raising the question of whether uniform IOP-lowering therapy is appropriate for all such eyes. However, no current evidence-based guidelines address the management of myopic glaucoma, and no long-term, prospective, multicenter, randomized controlled trial has evaluated the effect of IOP-lowering treatment on disease progression in myopic NTG.

To address this gap, we designed a long-term, prospective, multicenter, randomized controlled clinical trial—the Myopic Normal-Tension Glaucoma Treatment Study (MYNT-GTS)—that aims to determine the effect of IOP-lowering therapy on disease progression in patients with treatment-naïve myopic NTG presenting mild-to-moderate glaucomatous damage. The ultimate objective of this study is to establish clinical guidelines informed by disease pathophysiology that enable individualized and tailored therapeutic strategies for these patients. This article describes the study protocol and presents baseline characteristics of the enrolled participants, thereby serving as a foundation for future MYNT-GTS reports.

## 2. Materials and Methods

### 2.1. Study Design

**Figure 1:**
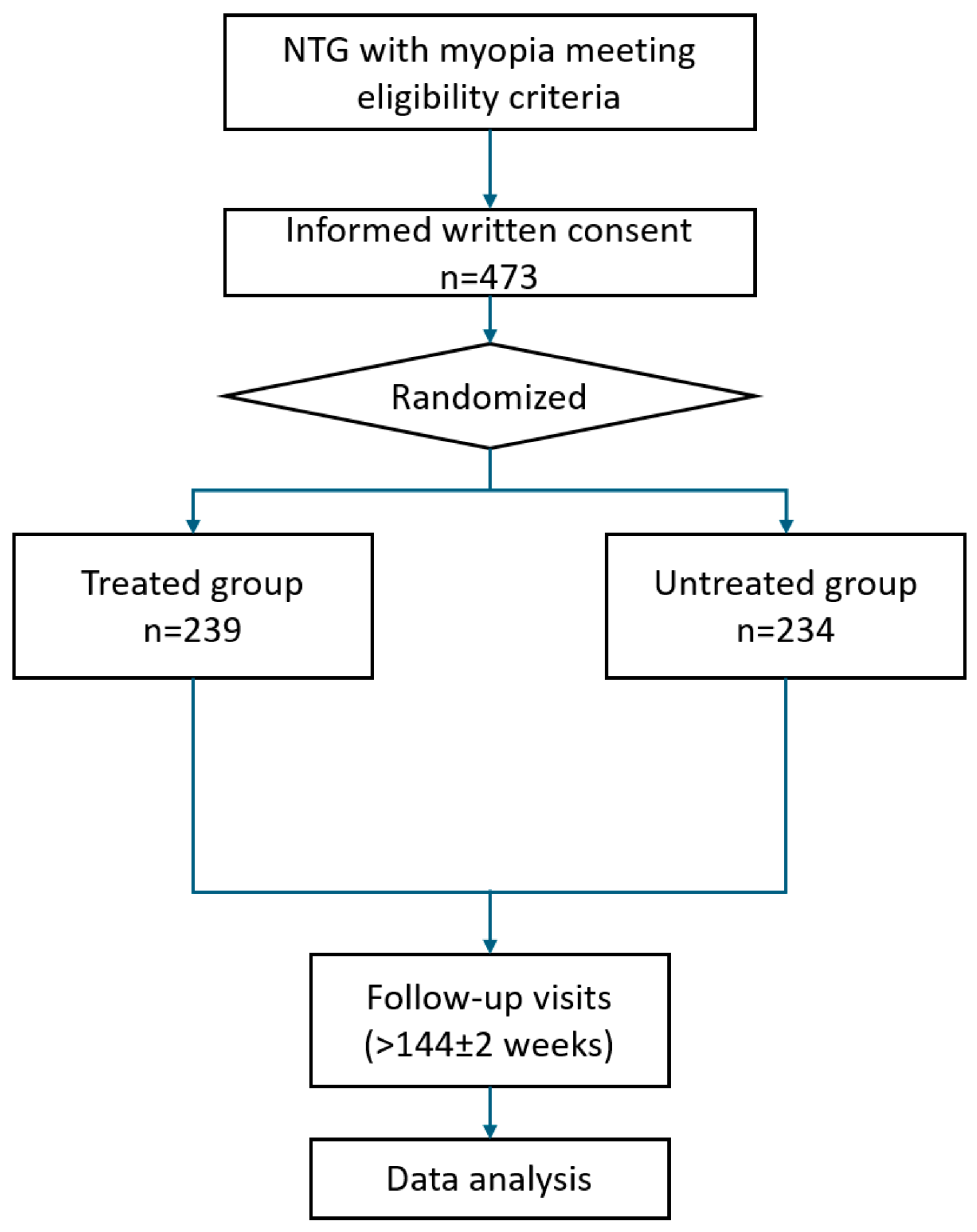
CONSORT flow diagram of participant screening, enrollment, and randomization in the MYNT-GTS (Myopic Normal-Tension Glaucoma Treatment Study). Of 498 patients screened, 21 were excluded since they did not meet the eligibility criteria and another 4 withdrew consent. The remaining 473 patients (1 eye in each) were enrolled and randomly assigned at a 1:1 ratio to the treated group (*n* = 239) and the untreated (active surveillance) group (*n* = 234). NTG, normal-tension glaucoma.

The MYNT-GTS is a prospective, multicenter, randomized controlled clinical trial. Participants were prospectively recruited from nine tertiary referral centers across South Korea: Seoul National University Bundang Hospital (SNUBH), Kangbuk Samsung Hospital (at Sungkyunkwan University), Pusan National University Hospital, Chonnam National University Hospital, Ewha Womans University Seoul Hospital, Gyeongsang National University Hospital, Samsung Medical Center (at Sungkyunkwan University), Kangwon National University Hospital, and Seoul National University Boramae Medical Center. Eligible patients were assigned to a treated group and an untreated group managed by active surveillance. The study adhered to the Declaration of Helsinki guidelines, and informed consent was obtained from all participants prior to their enrollment. The trial was prospectively registered with the Clinical Research Information Service (CRIS), Republic of Korea (KCT0005225; registered 13 July 2020; https://cris.nih.go.kr). This trial is reported in accordance with the Consolidated Standards of Reporting Trials (CONSORT) 2025 guidelines; the completed CONSORT 2025 checklist is provided as Supplementary Materials, and the participant flow is presented in Figure 1.

The primary goal of this study is to evaluate the impact of IOP-lowering therapy on disease progression in myopic NTG patients with mild-to-moderate glaucomatous optic neuropathy. The detailed objectives of this study are as follows:

1. To determine the effect of IOP-lowering treatment on glaucoma progression in patients with myopic NTG by comparing the glaucoma progression risk between untreated (active surveillance) and treated groups.
2. To determine the impact of structural characteristics of the ONH and peripapillary tissues on glaucoma progression.
3. To identify the individual characteristics of patients who show progression without IOP-lowering treatment versus those who experience rapid progression despite receiving treatment.
4. To characterize the long-term adherence to IOP-lowering treatment and its impact on the quality of life.

### 2.2. Screening Examinations

Participants underwent comprehensive screening examinations to determine their eligibility for inclusion in the study (V0 examinations, Supplemental Table S1). Medical-history evaluations included assessments of hypertension, diabetes mellitus, cold extremities, migraines, family history of glaucoma, previous medical and surgical history, current medications, and vital signs. Ophthalmic examinations encompassed best-corrected visual acuity assessments, Goldmann applanation tonometry, refraction tests, slit-lamp biomicroscopy, gonioscopy, fundus photography (Kowa VX-10α, Kowa Company, Nagoya, Japan), peripapillary retinal nerve fiber layer (RNFL) thickness measurements and enhanced depth-imaging scanning of the ONH using spectral-domain optical coherence tomography (OCT; Spectralis, Heidelberg Engineering, Heidelberg, Germany), OCT angiography (OCTA; DRI OCT Triton, Topcon, Tokyo, Japan) for evaluating the retinal and ONH microvasculatures, and standard automated perimetry (SAP; Humphrey Field Analyzer 3 and 24-2 Swedish Interactive Threshold Algorithm, Carl Zeiss Meditec, Dublin, CA, USA). Other ophthalmic examinations included measurements of the corneal curvature (KR-800, Topcon), central corneal thickness (CCT; Galilei G4 Dual Scheimpflug Analyzer, Ziemer Ophthalmic Systems AG, Port, Switzerland), and AXL (IOLMaster 700, Carl Zeiss Meditec). Examinations other than OCT, OCTA, SAP, and AXL measurement may have been performed using other devices at the discretion of the participating investigators.

### 2.3. Inclusion Criteria

The study included patients with treatment-naïve NTG aged 20–60 years with myopia. After screening, eyes that met the following criteria were enrolled: (1) spherical equivalent ≤ −1.0 D or AXL > 24.0 mm, (2) glaucomatous optic nerve damage confirmed in both optic disc and visual field (VF) examinations, and (3) untreated baseline IOP ≤ 21 mmHg. The untreated baseline IOP was taken as the average of two measurements obtained in the morning and afternoon at least 2 h apart; if the difference between the two measurements exceeded 2 mmHg, a third measurement was performed and the average of all three readings was used.

NTG was defined as the presence of an open iridocorneal angle and signs of glaucomatous optic nerve damage (i.e., neuroretinal rim thinning or notching, or an RNFL defect) accompanied by RNFL thinning in OCT and a glaucomatous VF defect at a location corresponding to the structural damage, with a maximum untreated IOP of ≤21 mmHg. The structural damage had to be distinguishable from nonglaucomatous myopia changes, such as apparent thinning or absence of neuroretinal rim—most often the temporal rim—caused by optic disc tilt and torsion, or diffuse myopia-related RNFL thinning that may produce false-positive OCT findings. A VF defect was defined as being glaucomatous when it met at least one of the following criteria: (1) outside the normal range in a glaucoma hemifield test, (2) three abnormal points with a < 5% probability of being normal and one with a < 1% probability according to the pattern deviation, or (3) a pattern standard deviation (PSD) probability of <5% confirmed on two consecutive reliable tests. These tests were considered reliable when the fixation-loss, false-positive, and false-negative rates were <20%, <15%, and <25%, respectively.

Eyes were excluded if they had a VF mean deviation (MD) worse than −12 dB, a glaucoma type other than open-angle glaucoma (e.g., angle-closure, secondary, or neovascular glaucoma), a history of ocular surgery (except uneventful cataract surgery, corneal refractive surgery, or intraocular lens implantation) or laser treatment (except a peripheral laser that does not affect the VF), a corneal disorder affecting IOP measurements, a history of ocular infection or trauma, or ocular diseases that may affect the ocular imaging quality (e.g., significant cataract or corneal disease) or VF reliability (e.g., retinal or neurological disease). Eyes with prior corneal refractive surgery were not excluded because such surgery is common among young adults with myopia in Korea, and excluding them would have substantially limited the representativeness of the study population. Their inclusion also allows the influence of prior refractive surgery, including its potential effect on IOP measurements, to be evaluated in subgroup and sensitivity analyses (Section 2.9). The study also excluded patients with a severe systemic disease that precludes ocular hypotensive therapy; a history of systemic or topical steroid or chemotherapeutic treatment during the preceding 60 days, or planned future treatment of this kind that could affect the optic nerve; or a psychiatric disorder severe enough to preclude completing questionnaires (e.g., dementia); as well as patients who were pregnant or considering pregnancy during the study period. The right eye was usually selected for inclusion when both eyes were eligible, although the left eye could be selected at the investigator’s discretion.

### 2.4. Randomization and Interventions

After obtaining informed consent from eligible patients, the finally enrolled participants were randomly assigned at a 1:1 ratio to an untreated (active surveillance) group and a treated group. Randomization was performed using a computer-generated random allocation table stratified by 4 predetermined variables to yield 16 strata: age (<40 and ≥40 years), baseline IOP (≤15 and >15 mmHg), AXL (≤26 and >26 mm), and VF MD (<−6 and ≥−6 dB).

Participants assigned to the untreated group received no initial IOP-lowering medications but were closely monitored for glaucoma progression. They were informed of the uncertainty regarding the treatment benefit in myopic NTG and were allowed to initiate treatment at any point upon progression detection. The treated group commenced receiving standard IOP-lowering eyedrops, with their efficacy assessed 1 month thereafter. First-line treatment was a topical prostaglandin analog (latanoprost) or a fixed combination of dorzolamide and timolol; when intolerance occurred or IOP reduction was insufficient, other commercially available topical ocular hypotensive agents were substituted or added.

Initiation, modification, or escalation of the IOP-lowering therapy was guided by two criteria: (1) the effectiveness of IOP reduction and (2) evidence of progressive glaucomatous change in structural or functional testing. Treatment was considered effective when at 1 month after initiation it had produced either (1) an IOP reduction of ≥20% or ≥3 mmHg relative to the baseline IOP, or (2) an absolute IOP of ≤9 mmHg. When these criteria were not met, the medication was changed or an additional agent was added, and the IOP-lowering effect was reassessed 1 month later using the same effectiveness criteria (see Section 2.5).

Structural progression was defined as occurring when the rate of circumpapillary RNFL thickness reduction—as determined by linear regression of serial spectral-domain OCT measurements—exceeded the 95th percentile of the rate observed in normal subjects [18,19,20]. Functional progression was defined as significant glaucomatous VF progression meeting the EMGT (Early Manifest Glaucoma Trial) criteria [21] in serial SAP. Whenever progression was detected in OCT or VF testing, IOP-lowering medication was newly prescribed in the previously untreated group or intensified in the treated group at the clinician’s discretion.

### 2.5. Follow-Up Schedule

Participants undergo comprehensive ophthalmic examinations at baseline and at scheduled follow-up visits, which occur every 4 months during the first year and every 6 months thereafter. These include measurements of best-corrected visual acuity, refractive error, IOP, corneal curvature, AXL, and CCT; optic disc photography; SAP; spectral-domain OCT; and OCTA (Supplemental Table S1). Quantitative analyses comprise optic disc tilt and torsion on disc photographs, peripapillary and ONH morphological parameters on OCT B-scans, and peripapillary vasculature deficits identified on OCTA images. Quality of life is also assessed using the Korean version of the National Eye Institute Visual Function Questionnaire-25 (NEI-VFQ-25) questionnaire.

When IOP-lowering therapy is initiated in either the treated or untreated group, an unscheduled visit is conducted 4 weeks later to evaluate its IOP-lowering effect. If the response is inadequate, the clinician can change the medication, add an additional agent, or perform a secondary intervention: selective laser trabeculoplasty or trabeculectomy. Whenever medication is modified or a secondary intervention is carried out during the study, another unscheduled visit is arranged 4 weeks later to reassess IOP control.

All study data are uploaded to a secure web-based server for review by the coordinating center (SNUBH). Data forms are examined for omissions and logical inconsistencies, with any queries sent to the applicable clinical sites for clarification or correction.

### 2.6. Compliance and Withdrawal Criteria

Each participant’s adherence to the prescribed interventions is assessed at each visit. A participant will be withdrawn from the study if any of the following circumstances occurs: poor adherence or cooperation that makes continued participation difficult, failure to complete the required follow-up period, an adverse event or other problem that makes continued participation harmful or impractical, withdrawal of consent, or any other circumstance in which the investigator judges that the participant is unable to continue.

### 2.7. Outcome Measures

The primary outcome is glaucoma progression as assessed from the rate of circumpapillary RNFL thinning and the rate of VF deterioration as assessed from the MD or visual field index (VFI). Glaucoma progression is thus a composite endpoint, deemed to have occurred when either the structural or the functional criterion (Section 2.4) is met. Secondary outcomes include factors correlated with the rates of RNFL thinning and VF deterioration. In addition, medication adherence and quality of life as assessed using the Korean version of the NEI-VFQ-25 questionnaire are evaluated as separate measures.

### 2.8. Sample Size

The required sample size was determined under a noninferiority framework, testing whether the proportion of eyes free of glaucoma progression over the observation period in the untreated (active surveillance) group was noninferior to that in the treated group. Assuming that IOP-lowering treatment had a protective effect in 80% of NTG patients (Collaborative Normal-Tension Glaucoma Study) [22], the proportions of participants who were free of progression were taken as 80.0% in the treated group and 75.2% in the untreated group, with the noninferiority margin of 15% being based on the US Food and Drug Administration guidance on noninferiority trials [23]. Achieving a one-sided α of 0.05 and 80% statistical power required 412 eyes (206 per group), and so the enrollment target was 474 eyes based on an assumed 13% loss to follow-up. The sample size was calculated using the method described by Chow et al. [24].

### 2.9. Data Analysis

Linear regression analyses are used to evaluate changes in RNFL thickness and VF indices over time. Kaplan–Meier survival analysis with log-rank testing is used to compare glaucoma progression rates between the untreated and treated groups. Longitudinal analyses will be performed using appropriate regression models while accounting for repeated measurements to assess the impact of initiating IOP-lowering therapy on progression rates among those patients originally assigned to active surveillance. Finally, multivariate multiple regression analyses will identify prognostic factors influencing disease progression, which will inform the development of a predictive scoring model. Central reading is performed to assess OCT image quality (including scan circle centering and segmentation errors) and VF reliability. Both intention-to-treat and per-protocol analyses will be performed. Eyes lost to follow-up will be censored at their last visit, and longitudinal data will be analyzed with mixed-effects models including center as a random effect. In the primary analysis, eyes in the untreated group in which treatment is initiated after progression are counted as events at the time of progression and analyzed in their assigned group; their subsequent follow-up is used only in the longitudinal analyses of the effect of initiating IOP-lowering therapy described above. Subgroup and sensitivity analyses will compare eyes with versus without prior corneal refractive surgery, which can lead to underestimation of IOP, and across axial-length strata.

## 3. Results

The recruitment phase took 50 months (from 20 October 2020 to 20 December 2024), which was longer than initially planned due to the COVID-19 pandemic. Of 498 patients screened, 21 were excluded since they did not meet the eligibility criteria and another 4 withdrew consent, leaving 473 patients enrolled. The patient enrollment process and randomization are shown in Figure 1; 239 and 234 patients were assigned to the treated and untreated groups, respectively.

Table 1 lists the baseline characteristics of the participants. Subjects enrolled in the MYNT-GTS were aged 43.5 ± 9.1 years (mean ± standard deviation), and their sex distribution was nearly balanced (239 males and 234 females). Most of the participants had normal systemic vital signs, with a systolic blood pressure (BP) of 124.5 ± 15.2 mmHg, diastolic BP of 75.9 ± 11.7 mmHg, body temperature of 36.7 ± 0.3 °C, and pulse rate of 78.8 ± 12.3 bpm. Their BMI was 23.8 ± 3.8 kg/m^2^.

Regarding lifestyle factors, 11.8% were current smokers, 14.2% were past smokers, and 74.0% had never smoked. Most (60.9%) were current alcohol drinkers, whereas 5.3% were past drinkers, and 33.8% reported never drinking. Comorbidities included diabetes mellitus in 6.6% of participants and systemic hypertension in 12.9%. Cold extremities and migraine were reported in 6.6% and 9.7% of participants, respectively. A family history of glaucoma in first-degree and second-degree relatives was identified in 19.2% and 7.4% of participants; the remaining 73.4% reported no known family history.

Regarding ophthalmic characteristics, the spherical equivalent was −4.45 ± 3.24 D, the AXL was 26.31 ± 1.36 mm, and the CCT was 528.9 ± 44.1 μm. The IOP at baseline was 14.8 ± 2.6 mmHg. The global RNFL thickness was 80.0 ± 12.3 μm. VF testing revealed an MD of −2.52 ± 2.55 dB, a PSD of 3.75 ± 2.83 dB, and a VFI of 94.7 ± 6.2%. Prior corneal refractive surgery had been performed in 28.5% of participants, and cataract surgery in 1.3%.

**Figure 2:**
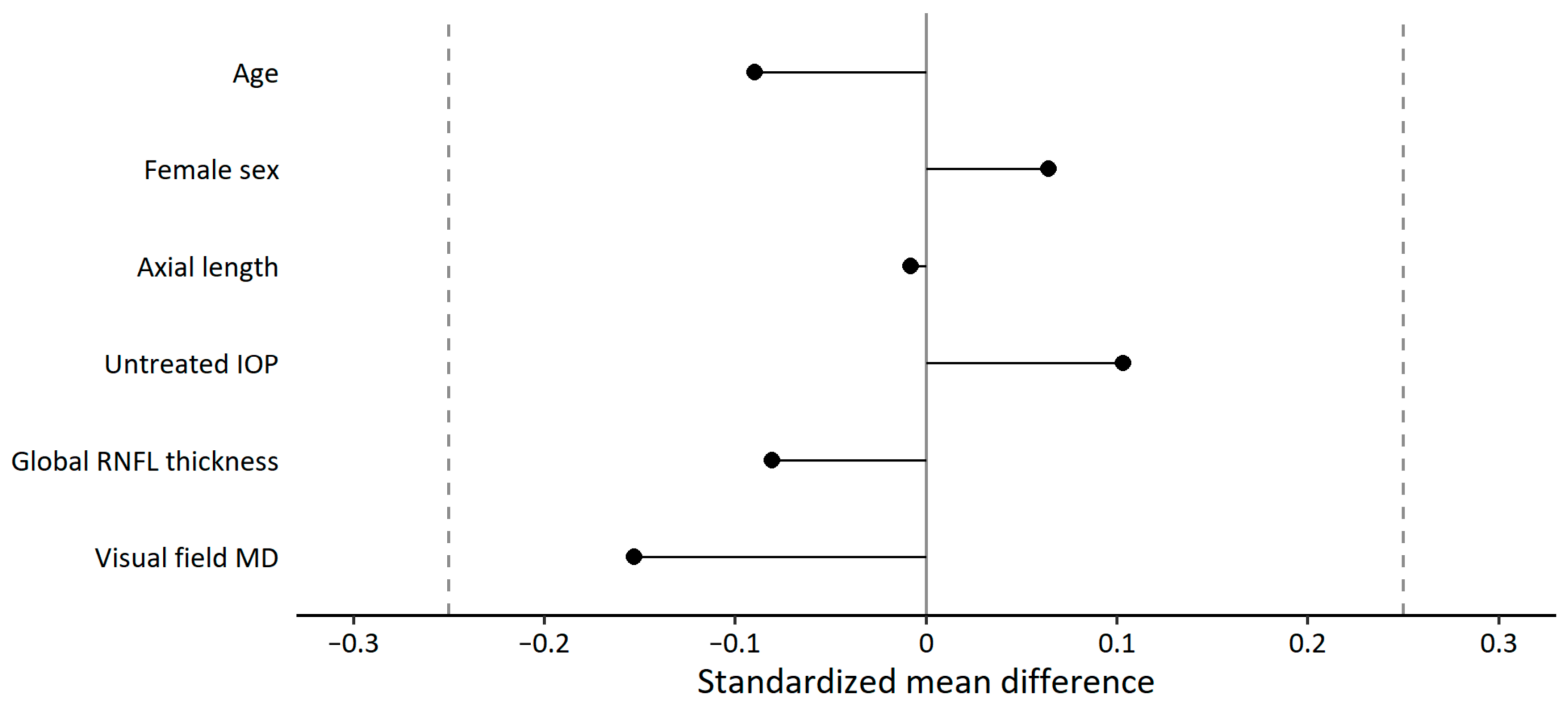
Plot of the standardized mean differences (SMDs) in baseline characteristics between the treated and untreated groups. Each dot represents the SMD (treated minus untreated) for a given baseline variable. The solid vertical line denotes an SMD of 0 (perfect balance), and the dashed lines delimit the range from −0.25 to 0.25, which is a commonly used criterion for acceptable between-group balance. All variables showed an absolute SMD of ≤0.16, indicating that the two groups were well balanced at baseline. IOP, intraocular pressure; RNFL, retinal nerve fiber layer; MD, mean deviation.

Randomization resulted in no significant differences in most clinical variables between the two groups; the exceptions were the systolic BP and VF indices (Table 1). The systolic BP was significantly lower (123.0 ± 15.0 vs. 126.1 ± 15.3 mmHg, *p* = 0.029) and the VF PSD was significantly higher (4.01 ± 2.88 vs. 3.49 ± 2.77 dB, *p* = 0.045) in the treated group than in the untreated group at the time of enrollment. The VF MD and VFI at enrollment were worse in the treated group than in the untreated group with marginal significance (−2.71 ± 2.58 vs. −2.32 ± 2.52 dB, *p* = 0.096; and 94.2 ± 6.3 vs. 95.2 ± 6.0%, *p* = 0.065; respectively). The standardized mean differences in the main baseline variables between the treated and untreated groups are shown in Figure 2; all were within the range from −0.25 to 0.25, confirming an acceptable baseline balance. Because significance testing after randomization has limited interpretive value, group comparability was assessed primarily using standardized mean differences (SMDs); the absolute SMDs of all baseline variables, including systolic BP and VF PSD, were ≤0.21.

## 4. Discussion

Myopic NTG is increasingly regarded as a distinct clinical context rather than a simple variant of typical NTG. Although myopia is predisposed to the development of glaucoma, the findings of several studies support that myopic NTG can progress more slowly than nonmyopic NTG, which complicates the application of uniform IOP targets and treatment intensities. However, no long-term, prospective, multicenter, randomized trial has determined whether IOP-lowering treatment alters progression in these eyes. We addressed this circumstance by designing the present prospective randomized study aimed at obtaining timely, evidence-based guidance. Participating clinical centers were selected from across South Korea based on three criteria: (1) excellence in glaucoma care, (2) demonstrated capacity to recruit eligible participants, and (3) established research capability and infrastructure. This protocol and the baseline cohort constitute the first dedicated framework focused specifically on myopic NTG.

Previous landmark randomized trials have established that IOP reduction is beneficial across the glaucoma spectrum, including NTG [22,25], early [21] and advanced [26] open-angle glaucoma, and ocular hypertensive disease [27], and a recent randomized trial addressed cases of suspected glaucoma with high myopia [28]. A long-term cohort study has also associated greater IOP reduction with less progression in highly myopic glaucoma [29]. However, none of those trials evaluated IOP-lowering treatment in established myopic NTG or related outcomes to the structural and vascular phenotypes of the myopia-specific ONH, and so the present trial was designed to address these gaps.

Beyond treatment comparisons, the MYNT-GTS incorporates detailed structural and microvascular phenotyping of the ONH, including LC morphology (e.g., the LC steepness index) [30], border-tissue configuration, and ONH and peripapillary microvasculatures. Such parameters have been used to predict VF progression in myopic NTG [31]. By relating these baseline phenotypes to subsequent progression, the study aims to identify mechanism-based markers that can identifying eyes that are likely to progress without treatment from those that will progress despite receiving treatment, thereby providing a foundation for the individualized, risk-stratified management of myopic NTG.

Because treatment decisions in myopic NTG may involve long periods of medication use in relatively young individuals, evaluating the treatment burden and quality of life was considered clinically important. The inclusion of the Korean version of the NEI-VFQ-25 therefore enables assessments of the patient-centered impacts of different management strategies.

A distinctive feature of the MYNT-GTS is the inclusion of an untreated (active surveillance) group. This design is supported by accumulating evidence that a substantial proportion of myopic NTG eyes remain stable without IOP-lowering treatment [6,7,8,9], which means that applying uniform treatment could expose many patients to the burden and adverse effects of therapy without providing any clear benefit. However, some treatment-naïve myopic NTG eyes progress within 2 years [32], underscoring the need for close monitoring. Because all untreated participants are closely monitored, with treatment initiated promptly once progression is detected, withholding initial treatment in the untreated group is ethically justifiable and directly addresses the central question of whether—and in whom—IOP-lowering treatment is necessary.

The randomization process successfully produced two groups with highly uniform characteristics in terms of demographic, general medical, and ophthalmic parameters; the exceptions were the systolic BP and the VF PSD. Despite these statistically significant intergroup differences, the systolic BP and VF PSD were essentially equivalent between the groups, because in a cohort of this size, even small, clinically negligible differences can reach statistical significance. In addition, the absolute differences were small: the differences of 0.39 dB in MD, 0.52 dB in PSD, and 1.0% in VFI all lie well within the known test–retest variabilities of SAP [33,34,35,36], whereas the 3.1-mmHg difference in systolic BP is well within the normal variability of clinic-based BP measurements [37]. Based on the reported association between systolic BP and NTG progression [38], the 3.1-mmHg difference would alter the risk of progression by only approximately 2 percentage points (assuming a 20% progression rate), which is small relative to the 15% noninferiority margin. Standardized effect sizes were also uniformly small (Cohen’s *d* ≤ 0.21; all within ±0.25, Figure 2), indicating the negligible magnitude of these discrepancies. Accordingly, the observed baseline imbalance is unlikely to translate into perceptible functional disparity or to confound subsequent treatment effects. We therefore interpret the groups as being clinically equivalent at study entry, supporting the internal validity of our outcome comparisons.

The previous randomized trial that is most directly relevant to the present study is the GSHM (Glaucoma Suspects with High Myopia) study, which is a single-center trial testing whether medically lowering IOP reduces progression in patients with suspected highly myopic glaucoma [28]. The MYNT-GTS differs from that previous trial in several important respects. First, it has enrolled patients with established myopic NTG rather than those with suspected glaucoma, thereby examining the effect of lowering IOP on progression once glaucomatous damage is already present. Second, it includes a broader spectrum of myopia rather than only high myopia. Third, its multicenter design across nine tertiary referral centers supports broader generalizability. The MYNT-GTS also incorporates structural and microvascular phenotyping of the ONH, allowing examination of how these features relate to progression. The two trials thus address complementary parts of the myopic glaucoma spectrum, and their combined findings may help clarify when—and in whom—IOP-lowering treatment is warranted in myopic eyes.

Several limitations of the MYNT-GTS should be acknowledged. First, all participants are Korean, which may restrict the ability to generalize the findings to populations with different ancestries, refractive patterns, or healthcare systems. Nevertheless, the high prevalence of NTG in Korea [1] made this focused, prospective evaluation feasible. Second, eyes with prior corneal refractive surgery were included, which may have introduced heterogeneity and could influence IOP measurements and structural metrics. However, this inclusion does allow subgroup and sensitivity analyses comparing eyes with and without refractive surgery in future work. Third, although the first-line treatment was standardized, the escalation and choice of additional IOP-lowering agents is left to the discretion of the treating clinician, which may introduce some variability in the applied treatments. This pragmatic approach nonetheless reflects real-world clinical practice and aligns with the study’s aim of informing individualized therapeutic strategies. Fourth, the progression rates assumed in the sample size calculation were derived from the Collaborative Normal-Tension Glaucoma Study [22], which was not limited to myopic eyes; however, the lower progression rates expected in myopic NTG would increase, rather than decrease, the statistical power. The 15% noninferiority margin may be considered lenient for an irreversible outcome. For example, if the progression-free proportions were 90.0% and 85.2% rather than assumed 80.0% and 75.2%, respectively, the statistical power would increase from 80% to approximately 93%. However, the 15% margin was selected to balance clinical acceptability with study feasibility, considering that participants were closely monitored and treatment was promptly initiated upon confirmed progression. Finally, the open-label design—necessitated by the comparison of treatment versus active surveillance—precludes the masking of participants and treating clinicians, and so may introduce bias.

An important function of the MYNT-GTS will be to refine guidelines for tailored treatment applied to myopic NTG patients: informing target IOP ranges, treatment intensities, and follow-up schedules based on individualized risk profiles. The present report has provided the protocol and the baseline cohort that will serve as the foundation for subsequent MYNT-GTS outcome analyses and future reports.

## Figures and Tables

**Table 1:** Baseline demographic and ophthalmic characteristics.

	Overall Participants (*n* = 473)	Treated Group (*n* = 239)	Untreated Group (*n* = 234)	*p*-Value
**Demographic characteristics**				
Age, years	43.5 ± 9.1	43.1 ± 9.1	43.9 ± 9.1	0.328
Gender, male/female	239/234	117/122	122/112	0.489
Systolic blood pressure, mmHg	124.5 ± 15.2	123.0 ± 15.0	126.1 ± 15.3	0.029
Diastolic blood pressure, mmHg	75.9 ± 11.7	75.1 ± 11.8	76.8 ± 11.5	0.113
Body temperature, °C	36.7 ± 0.3	36.7 ± 0.3	36.7 ± 0.3	0.536
Pulse rate, bpm	78.8 ± 12.3	79.1 ± 11.7	78.4 ± 13.0	0.522
Body mass index, kg/m^2^	23.8 ± 3.8	23.7 ± 4.0	23.9 ± 3.6	0.535
Smoking, *n* (%)				0.243
Current	56 (11.8%)	29 (12.1%)	27 (11.5%)	
Past *	67 (14.2%)	40 (16.7%)	27 (11.5%)	
Never	350 (74.0%)	170 (71.1%)	180 (76.9%)	
Drinking, *n* (%)				0.576
Current	288 (60.9%)	142 (59.4%)	146 (62.4%)	
Past ^†^	25 (5.3%)	15 (6.3%)	10 (4.3%)	
Never	160 (33.8%)	82 (34.3%)	78 (33.3%)	
Diabetes mellitus, *n* (%)	31 (6.6%)	14 (5.9%)	17 (7.3%)	0.536
Systemic hypertension, *n* (%)	61 (12.9%)	32 (13.4%)	29 (12.4%)	0.747
Cold extremity, *n* (%)	31 (6.6%)	14 (5.9%)	17 (7.3%)	0.536
Migraine, *n* (%)	46 (9.7%)	25 (10.5%)	21 (9.0%)	0.586
Family history, *n* (%) First degree relatives Second degree relatives None	91 (19.2%) 35 (7.4%) 347 (73.4%)	51 (21.3%) 21 (8.8%) 167 (69.9%)	40 (17.1%) 14 (6.0%) 180 (76.9%)	0.206
**Ophthalmic characteristics**				
Corneal refractive surgery, *n* (%)	135 (28.5%)	74 (31.0%)	61 (26.1%)	0.239
Cataract surgery, *n* (%)	6 (1.3%)	3 (1.3%)	3 (1.3%)	0.979
Spherical equivalent, D	−4.45 ± 3.24	−4.35 ± 3.24	−4.55 ± 3.24	0.512
Axial length, mm	26.31 ± 1.36	26.31 ± 1.37	26.32 ± 1.34	0.935
Central corneal thickness, μm	528.9 ± 44.1	528.0 ± 45.2	529.8 ± 43.0	0.657
Intraocular pressure, mmHg	14.8 ± 2.6	14.9 ± 2.6	14.7 ± 2.5	0.263
Global RNFL thickness, μm	80.0 ± 12.3	79.5 ± 11.4	80.5 ± 13.2	0.379
Visual field MD, dB	−2.52 ± 2.55	−2.71 ± 2.58	−2.32 ± 2.52	0.096
Visual field PSD, dB	3.75 ± 2.83	4.01 ± 2.88	3.49 ± 2.77	0.045
Visual field VFI, %	94.7 ± 6.2	94.2 ± 6.3	95.2 ± 6.0	0.065

bpm = beats per minute; D = diopters; MD = mean deviation; PSD = pattern standard deviation; RNFL = retinal nerve fiber layer; VFI = visual field index. * a participant who had previously smoked (including conventional or electronic cigarettes) but had stopped by the time of enrollment. ^†^ a participant who had previously consumed alcohol but had stopped by the time of enrollment.

## Data Availability

All relevant data underlying the findings are available in the iCReaT (Internet-based Clinical Research and Trial Management System) repository of the Korea National Institute of Health (https://icreat.nih.go.kr/icreat2/invoke/CM000000/ (accessed on 1 October 2026)). Additional de-identified data are available from the corresponding author on reasonable request, subject to institutional review board approval.

## Supplementary Materials

The following supporting information can be downloaded at: https://www.mdpi.com/article/10.3390/jcm15197734/s1, Table S1: Scheduled Clinical Evaluations and Follow-Up Visits. The completed CONSORT 2025 checklist is provided as Supplementary Materials. Reference [39] are cited in the Supplementary Materials.
