# Supplementary material for: The Myopic Normal Tension Glaucoma Treatment Study (MYNT-GTS): Protocol and Baseline Characteristics of a Randomized Controlled Trial
Source: J Clin Med. 2026 Oct 8;15(19):7734. doi: 10.3390/jcm15197734 (PMC13666113; doi:10.3390/jcm15197734)
Supplement: Supplementary file 1 [file jcm-15-07734-s001.zip › jcm-4607948-supplementary.pdf]

Table S1. Scheduled Clinical Evaluations and Follow-Up Visits[1]

Assessment		Visit 0 (Screening)	Visit 1 (16±2wk)	Visit 2 (32±2wk)	Visit 3 (48±2wk)	Visit 4 (72±2wk)	Visit 5 (96±2wk)	Visit 6 (120±2wk)	Visit 7 (144±2wk)	Unscheduled Visit
Informed consent / screening number assignment[2]		●								
Demographics[3]		●								
Inclusion/exclusion criteria		●								
Ophthalmic diagnosis[4]		●								
Medical history[5]		●								
Quality of life questionnaire (NEI-VFQ-25)[6]		●	○	○	○	○	●	○	○	○
Surgical and medical history[7]		●								
Concomitant medications[8]		●	●	●	●	●	●	●	●	●
Adverse events[9]			●	●	●	●	●	●	●	●
Vital signs[10]		●			○		○		○	
**General ophthalmic examinations**[11]	Best-corrected visual acuity[12]	●			●		●		●	
	Corneal curvature, axial length, and central corneal thickness	●							●	
	Slit-lamp biomicroscopy	●								
**Glaucoma diagnostic examinations**[13]	Intraocular pressure[14]	●	●	●	●	●	●	●	●	●
	Optic nerve head assessment	●			●		●		●	
	Retinal nerve fiber layer assessment	●			●		●		●	
	Standard automated perimetry	●(×2)	●(×2)	●	●	●	●	●	●	
	Spectral-domain OCT	●(×2)	●	●	●	●	●	●	●	
	Lamina cribrosa parameters	●	●	●	●	●	●	●	●	
	Fundus photography and OCT angiography	●			●		●		●	
Randomization[15]		●								
IOP-lowering protocol (treated group)[16]		●								
Intervention summary[17]			●	●	●	●	●	●	●	
Change or addition of glaucoma therapy			●	●	●	●	●	●		●
Reasons for withdrawal			●	●	●	●	●	●	●	●

●, performed; ○, also performed at the indicated visits (see footnotes); ×2, performed twice.

wk, weeks; OCT, optical coherence tomography; IOP, intraocular pressure; NEI-VFQ-25, National Eye Institute Visual Function Questionnaire-25.

[1] Visits 1–7 are scheduled relative to Visit 0 (Screening) with an allowed window of ±2 weeks; the screening number and the randomization number are assigned on the same day. In both groups, an unscheduled visit is performed 4 weeks later whenever IOP-lowering treatment is started, a medication is added or changed, or a secondary (laser or surgical) treatment is performed, to evaluate the IOP-lowering effect.

[2] The screening number is assigned in the order in which voluntary written informed consent is obtained, before any screening procedure.

[3] Demographic information (initials, date of birth, age, sex) and drinking/smoking history are collected.

[4] The glaucomatous (study) eye and the date of initial diagnosis are recorded.

[5] Diabetes, hypertension, cold extremities, migraine, and family history of glaucoma are assessed.

[6] Performed with the Korean version of the National Eye Institute Visual Function Questionnaire-25 (NEI-VFQ-25; J Korean Ophthalmol Soc 2010;51(10):1354–1367).

[7] Past and current medical history, including any surgical history within 1 year before screening, is collected; corneal refractive surgery history is collected over the entire lifetime.

[8] Medication history within 30 days before screening is collected (within 60 days for glaucoma medications and steroid eye drops). From Visit 1, only changes since the previous visit are recorded.

[9] Adverse events are reported voluntarily by participants and ascertained by the investigator at scheduled and unscheduled visits.

[10] At Visit 0, height, weight, body-mass index, body temperature, blood pressure, pulse, and waist/hip circumference are measured. Blood pressure and pulse are also measured at Visits 3, 5, and 7 (marked ○).

[11] May be substituted by results obtained within 2 months before the consent date (within 6 months for axial length and central corneal thickness), at the investigator's discretion.

[12] Measured with a Snellen chart; if another chart is used, values are converted to Snellen equivalents.

[13] May be substituted by results obtained within 2 months before the consent date, at the investigator's discretion.

[14] IOP is measured twice and the average is used; if the difference exceeds 2 mmHg, a third measurement is taken and the average of all three is used.

[15] After eligibility is confirmed, participants are randomly assigned to the treated or untreated group using a computer-generated random allocation table, in a 1:1 ratio.

[16] Participants in the treated group start IOP-lowering treatment immediately; those in the untreated group undergo active surveillance and start treatment only if progression occurs. Treatment is considered effective when, one month after initiation, IOP shows a ≥20% or ≥3 mmHg reduction from baseline or an absolute value ≤9 mmHg; otherwise medication is changed/added or secondary treatment (selective laser trabeculoplasty or trabeculectomy) is performed, with reassessment 4 weeks later.

[17] All current first- and second-line treatments are recorded.
